# The No-Report Paradigm: A Revolution in Consciousness Research?

**DOI:** 10.3389/fnhum.2022.861517

**Published:** 2022-05-11

**Authors:** Irem Duman, Isabell Sophia Ehmann, Alicia Ronnie Gonsalves, Zeynep Gültekin, Jonathan Van den Berckt, Cees van Leeuwen

**Affiliations:** ^1^Brain and Cognition, Faculty of Psychology and Educational Sciences, KU Leuven, Leuven, Belgium; ^2^Cognitive and Developmental Psychology, Faculty of Social Sciences, TU Kaiserslautern, Kaiserslautern, Germany

**Keywords:** consciousness, awareness, critique of methodology, perceptual ambiguity, binocular rivalry, introspection

## Abstract

In the cognitive neuroscience of consciousness, participants have commonly been instructed to report their conscious content. This, it was claimed, risks confounding the neural correlates of consciousness (NCC) with their preconditions, i.e., allocation of attention, and consequences, i.e., metacognitive reflection. Recently, the field has therefore been shifting towards no-report paradigms. No-report paradigms draw their validity from a direct comparison with no-report conditions. We analyze several examples of such comparisons and identify alternative interpretations of their results and/or methodological issues in all cases. These go beyond the previous criticism that just removing the report is insufficient, because it does not prevent metacognitive reflection. The conscious mind is fickle. Without having much to do, it will turn inward and switch, or timeshare, between the stimuli on display and daydreaming or mind-wandering. Thus, rather than the NCC, no-report paradigms might be addressing the neural correlates of conscious disengagement. This observation reaffirms the conclusion that no-report paradigms are no less problematic than report paradigms.

## Introduction

When you are conscious, you are always conscious of something. Being conscious is often associated with states such as “being awake”, and “being responsive” as opposed to “anesthetized,” or “comatose”. State-based, or intransitive, characterizations of consciousness may be useful for clinical purposes, but they can also be confusing; see the debate about whether persistent vegetative state patients can be conscious (Owen et al., 2006). In particular, such definitions are rather uninformative about what it means to be conscious of something, also known as transitive consciousness (Rosenthal, 2009). When analyzing transitive consciousness, Block (1995, 2005) distinguishes *phenomenal* and *access consciousness*. Whereas phenomenal consciousness refers to a person having a certain sense of something, namely a sense that comes with a distinct, subjectively experienced quality, access consciousness is the availability of sensed content for use in processes such as reasoning and action (Block, 1995).

The dual aspects of transitive consciousness, subjective experience, and meaningful content, also play a key role in a recent attempt at a definition by Pereira and Ricke (2009). According to their definition, the requirements for consciousness encompass three elements: content, a person to experience the content as being meaningful, and a link of the content with the person’s behavior (p. 43). Content can be sensed without being experienced (e.g., blindsight, skillful automatic behaviors when driving a car); conversely experience may sometimes be characterized by absence, severely degraded, or diminished content (vegetative state, speaking in tongues, delusions, etc.). But in everyday vanilla consciousness, subjective experience and content go hand in hand. Let that be our working characterization of consciousness: a degree of both will have to be ascribed to a person to call them conscious.

### Neural Correlates of Consciousness

Whereas consciousness is typically ascribed to a person, cognitive neuroscience believes it to originate at the subpersonal level. Opinions differ on almost every aspect of how these levels are connected. Few researchers would doubt, however, that the neural substrate must be at the core of subpersonal level explanation (Sterzer et al., 2009). Koch (2018) defines the *neural correlates of consciousness* (NCC) as the “minimal neuronal mechanisms jointly sufficient for any specific conscious experience”. By speaking of “specific conscious experience”, this approach aims to distinguish NCC from general enabling factors, such as the presence of certain factors regulating arousal that are necessary for a conscious state, e.g., wakefulness (Mormann and Koch, 2007). We may think of enabling conditions as state consciousness and its prerequisites, whereas NCC regards transitive consciousness.

By speaking of “correlates”, the definition of NCC is careful to avoid any commitment to doctrines of mind-brain relationship such as dualism (mechanisms that cause or produce consciousness), supervenience (mechanisms that instantiate consciousness), or identity theory (mechanisms that constitute consciousness). By emphasizing correlates over causes, the definition undervalues research contributions using brain stimulation to influence conscious contents (Selimbeyoglu and Parvizi, 2010). The NCC program is focused on research paradigms that allow us to infer the NCC from the neural signals observed in experiments using a range of available neuroimaging techniques, such as functional magnetic resonance imaging (fMRI), electroencephalography (EEG), or magnetoencephalography (MEG), and a range of invasive techniques, mostly for use in nonhuman animals (Gamez, 2014). However, there is a good degree of overlap between the methods used in both (Kim and Blake, 2005; Tong et al., 2006).

The concept of NCC purports to be neutral with respect to theories of consciousness. However, its definition is less neutral than it seems. To begin with, it speaks of “jointly sufficient” mechanisms. This phrase implies that consciousness is an intrinsic property of certain brain states. This excludes a notion of consciousness as a product of contextual emergence, in which some but not all of the conditions for a higher-level phenomenon exist at a lower level (Bishop and Atmanspacher, 2006; Jordan and Ghin, 2006; van Leeuwen, 2007). Contextual emergence regards consciousness as a synergistic phenomenon different from its interacting component mechanisms, driven, at least in part, by dynamics at the level of the person and its interaction with its environment, exercising downward causality on its components. Contextual emergentism provides a natural framework for, for instance, theories that view consciousness at the personal level as grounded in learning (Cleeremans et al., 2020), coordination behavior (Solfo and van Leeuwen, 2018), or from neural prediction failure (e.g., Solms and Friston, 2018; Luczak and Kubo, 2022), Adopting the notion of NCC as defined puts these views in a reductionist straightjacket.

Moreover, the definition speaks of “minimal” jointly sufficient neural mechanisms. This suggests that a limited set of core mechanisms should be isolated. It follows that certain neural mechanisms are redundant, or spurious correlates of consciousness (Aru et al., 2012; de Graaf et al., 2012). de Graaf et al. (2012) distinguish between neural prerequisites and neural consequences of conscious experience. Prerequisites may elicit, facilitate, or moderate an NCC but are not necessary for consciousness to occur. Neural consequences of consciousness, in turn, could be elicited, facilitated, or moderated by NCC but the occurrence of consciousness does not depend on that.

The minimalism of Koch (2018) is poised to consider attention a prerequisite, rather than a genuine NCC. Koch and Tsuchiya (2007) emphasize that there are instances where consciousness occurs without a need for top-down attention (i.e., priming). We find this less than convincing. Priming also occurs without consciousness, e.g., in masked priming experiments (Forster and Davis, 1984, but see Holender, 1986 for a critique of this understanding). This double dissociation might suggest that priming is an independent mechanism that only incidentally is associated with consciousness (e.g., it involves the “content” but not necessarily the “experience” component of consciousness).

Mack and Rock (1998) argued that consciousness cannot occur without attention. A conspicuous item in a scene may be missed if attention is occupied elsewhere. A famous illustration of this *in attentional blindness* phenomenon is the “invisible gorilla” (Simons and Chabris, 1999). Participants were instructed to watch a video of a basketball game and count the passes made by a certain team. At some point in the video, a person wearing a gorilla costume walked through the scene. Interestingly, half of the participants did not notice the gorilla as their attention was occupied with counting the passes.

The role of attention in consciousness has led Graziano and Kastner (2011) to propose the Attention Schema Theory (AST). AST views subjective consciousness (referred to as awareness) as a simplified model of attention. It suggests that awareness is a reconstruction of our attentional processes that facilitates our control over them. During this process of reconstruction, the attention schema leads the brain to think that it has a subjective experience of stimuli (Graziano and Webb, 2015). Let this be sufficient to illustrate that opinions differ on whether attention is a mere prerequisite of consciousness, or even whether trying to eliminate it from your studies is beneficial.

Koch’s (2018) minimalism is likewise biased to consider certain processes as neural consequences of consciousness rather than NCC which others have considered necessary, such as judging a stimulus, keeping their answer in working memory, and deciding on a response. Some of these processes have introspective and meta-cognitive components. Introspection involves observation and examination of one’s own emotional and mental processes, metacognitive processes involve cognitive reflections on subjective experiences (Michel, 2017). Higher-order theories argue that consciousness crucially depends on processes representing oneself as being in a particular mental state (Lau and Rosenthal, 2011). According to Shea and Frith (2019), introspective and metacognitive processes are essential for presenting information in an integrated format global required for access to the global workspace, the purported “theatre of consciousness” (Baars, 1988).

In sum, Koch’s minimalism directs the research focus on a rather narrow set of neural mechanisms and induces researchers to disentangle them from a set of closely related ones. This even includes processes other researchers consider essential to consciousness. Koch’s project is one of high stakes and long odds, which has had quite considerable resonance recently. Perhaps, some ten years later, it is time to take stock, to see what this proposal has brought us.

### Paradigms for Investigating Consciousness

For observing NCC, two experimental design strategies exist in principle: direct and indirect approaches (Overgaard, 2017). Direct approaches involve self-reports of subjective experience, and are collectively labeled “report-based paradigms”. Indirect approaches refer to measurements that seek to infer experience from behavioral or brain data rather than subjective reports and are, thereby, referred to as the “no-report paradigms” (Tsuchiya et al., 2015).

It might seem intuitive to rely on verbal or behavioral reports to ensure subjects were conscious of a specific stimulus as these subjective experiences are only directly available to those who experience them (Tsuchiya et al., 2015; Fazekas and Overgaard, 2018). We need such first-person evidence of an experience, it might appear, to associate it with some neural signal. Yet, the need to report conscious content may invoke neural prerequisites and consequences, in addition to NCC (Aru et al., 2012; Tsuchiya et al., 2015). For instance, in order to report a conscious experience, it may be necessary to invest attentional resources in a stimulus (Koch and Tsuchiya, 2007; Tsuchiya and Koch, 2014), thereby invoking a neural prerequisite. The report will also invoke introspective and metacognitive processes. The neural mechanisms behind these processes might qualify as neural consequences (Aru et al., 2012; de Graaf et al., 2012). In a report paradigm, these supposedly spurious correlates would always co-occur with the NCC, and hence it would be impossible to disentangle them from the NCC (Michel, 2017). Report-based paradigms would, therefore, lack the specificity needed to identify the minimal set of processes related to the subjective experience.

Thus, the major impetus by researchers such as Koch and Tsuchiya has been to drive researchers away from report paradigms. No-report paradigms opt to study NCC by eliminating self-report (Block, 2019). No-report paradigms, therefore, face the problem of how to establish conscious experience without first-person evidence. Researchers have found elegant solutions to these problems. According to Block (2019), these paradigms do not exclude metacognitive reflection. We will discuss specific examples of no-report paradigms and analyze their results. Our analysis will mainly focus on a number of studies that have been brought forward to demonstrate the merit of no-report paradigms. As we will observe, these studies often have alternative explanations and face methodological problems. The more general of these problems goes beyond the earlier criticisms by Block (2019) and, in fact, also apply as well to his proposed solution.

### Report-Based Paradigms

Traditionally, research investigating the neural basis of consciousness relied on report-based paradigms (Michel, 2017). A typical paradigm of this kind should allow differentiating changes in conscious experience from neural events associated with changes in the environment. A popular paradigm uses ambiguous stimuli (e.g., the Necker cube); this allows that the stimulus is kept physically constant, while the conscious experience varies over time (de Graaf et al., 2012; Tsuchiya et al., 2015). We may distinguish between onset-ambiguity paradigms, in which observers report their percept following short exposures of the stimulus at different trials (Kubovy and Wagemans, 1995; Nikolaev et al., 2008; Kornmeier and Bach, 2012) and perceptual switching paradigms, allowing perceptual switching during prolonged exposure to a stimulus (Einhäuser et al., 2004; Nakatani and van Leeuwen, 2006). In both paradigms, the sequence of conditions in the experiment is defined by the participant’s response and not, as usual, determined by the experimenter (de Graaf et al., 2012).

In onset-ambiguity paradigms, the neural activity of the participant is aligned with stimulus onset, and event-related components are extracted. These are classified according to the report, in order to determine those that covary with the conscious experience. This procedure facilitates the extraction of early evoked components of the brain signal.

Nikolaev et al. (2008) used an onset paradigm with ambiguously oriented dot lattices as stimuli, with different degrees of ambiguity based on the Gestalt principle of grouping by proximity. Two early components (C1 and P1) were associated with the response; only the first one positively correlated with the proximity principle.

In a subsequent examination of the data by Nikolaev et al. (2008), Nikolaev et al. (2016) observed that responses unrelated to proximity were informed by intrinsic perceptual bias, such as a preference for the vertical orientation. The authors concluded that such biases determine the response when working memory operations intermittently weaken the first signal (C1). A regulatory, attentional mechanism dynamically moderates the responses, depending on the relative strength of the evoked components. This mechanism is revealed by fluctuations in the strength of an ongoing alpha component (Nikolaev et al., 2016).

We may wonder, whether any of the evoked components, C1 or P1, or the ongoing alpha in isolation would uniquely be responsible for consciousness. Rather, it is the particular way in which they dynamically interact that determines the sequence of conscious responses in its entirety. If this were the rule, it would mean that we cannot isolate any spurious correlates as prerequisites or consequences of NCC. The sequence of conscious reports is better described as emergent from the interplay of sensory perception, perceptual bias, working memory, and regulatory mechanisms for the allocation of attention.

**Figure 1:**
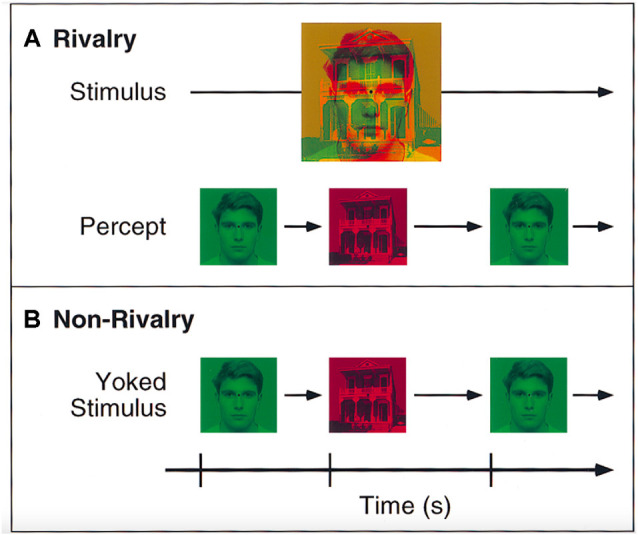
Illustration of the Binocular Rivalry task. **(A)** Depiction of two different stimuli (i.e., face and house). Each is presented simultaneously to one eye, which leads to binocular rivalry as indicated by differences in perception. **(B)** In contrast, illustration of a yoked stimulus (each stimulus is matched to the rivalry condition). This condition involves the presentation of the same stimuli consecutively without rivalry. Reprinted with permission from the publisher of “Binocular Rivalry and Visual Awareness in Human Extrastriate Cortex” by Tong et al. (1998). Copyright (1998) by Cell Press.

A switching paradigm is typically used to study binocular rivalry (Doesburg et al., 2005). As illustrated in Figure 1, an ambiguity is created by presenting rivaling stimuli in each eye. Typically, only one of the simultaneously presented stimuli is consciously perceived. Which of the rivaling stimuli is presently in consciousness is reported through a button press. Perceptual switches are contrasted with a replay, in which the same stimuli are presented yoked to both eyes. This contrast enables a focus on mechanisms controlling *spontaneous* switches in perception, as opposed to changes in the stimulus. Switches were found to be correlated with short bursts of increased global Gamma-(30–50 Hz) band phase synchrony in EEG, which are absent prior to the onset of the perceptual event (Doesburg et al., 2005). The increase in the global **γ**-band phase may be a neural correlate of perceptual consciousness. The effect is similar to that found with switches in ambiguous figures like the Necker cube (Nakatani and van Leeuwen, 2006).

In the switching paradigm, event-related signals are typically aligned with the report of the switch. It takes time for the switch to occur, so prolonged exposure to the stimuli is needed. This means that the role of oculomotor behavior needs to be taken into account. In studies with the Necker cube, eye position (Einhäuser et al., 2004), as well as saccade direction (Nakatani et al., 2012) and blink timing (Nakatani and van Leeuwen, 2013; Brych et al., 2021), are shown to moderate perceptual switches. This further suggests that conscious reports cannot be reduced to a minimal set of isolated neural events, but instead relate to a dynamic scenario. In ambiguous figures, early processes are laying out multiple interpretations (Nikolaev et al., 2008), overt and covert attentional processes bias the selection (Nakatani et al., 2012), and moderate the switching and the role of gamma bursts in it (Nakatani and van Leeuwen, 2006). A conscious event like switching cannot be reduced to gamma burst, for instance, as the only genuine NCC, but spans a much longer and complex dynamical process.

The examples discussed illustrate, on the one hand, that report paradigm data can be explained in terms of a dynamic synergy of a broad range of component mechanisms. This may be welcomed as a case in point for contextual emergentism. On the other hand, it would not impress the protagonists of minimalist NCC, who understand such a conclusion as a mere illustration of how confounded report paradigms are.

### No-Report Paradigms

No-report paradigms rely on eye-movement, neuro-imaging, or physiological measures as indicators of consciousness. In the context of visual perception, measures of eye movements or pupil size are particularly useful. Researchers have shown considerable inventiveness in devising such measures. Ideally, they are first validated using the report paradigm and are then used to infer perceptual contents, thereby functioning as “perceptual readouts” in no-report conditions (Overgaard and Fazekas, 2016; Block, 2019).

#### No-Report Flash Suppression

Wilke et al. (2009) conducted a study in monkeys using an onset ambiguity task called generalized flash suppression. A salient target stimulus (a red disk) was presented, followed by the sudden onset of a moving random dot pattern. Under well-established conditions, the target disappears completely for several seconds. Precise calibration of the conditions allows stimuli to be ambiguous, in the sense that the disk disappears about half of the time. The main experiment followed the procedure of a report paradigm. The monkeys had been trained long hours with non-ambiguous stimuli to report whether the disk had disappeared, and trials were sorted accordingly. Recording targeted neuronal spiking as well as population behavior in LGN and the pulvinar. In the latter area, effective flash suppression had approximately the same effect on neuron spiking as physically removing the target stimulus from the screen, whereas it left the firing of LGN neurons unaffected. This result suggests that selection does not arise early in the visual system.

Whereas these results are obtained with a report paradigm, in a control experiment monkeys were exposed to passive viewing. This offers us a first occasion to see, to what extent a no-report paradigm is able to eliminate spurious correlates and disentangle NCC. In this condition, however, no ambiguous stimuli could be used. Instead, the likelihood of suppression was determined by using unambiguous stimulus configurations, each of which definitely either does or does not lead to suppression.

As far as neuronal spiking was concerned, the effects in the control experiment were no different from the main experiment. However, neuronal population behavior as recorded by local Field Potentials (LFP) showed a remarkable difference between report and no-report experiments. LFP represents summed electrical potential from a population of neighboring neurons (Sharott, 2014). It is believed to reflect synaptic activity or input received by the observed area (Einevoll et al., 2013; Tsuchiya et al., 2015). In the report condition, reduction of power in the alpha and beta frequency bands (9–30 Hz) of the LFP was observed in the pulvinar in association with perceptual suppression. However, no such effect was observed in the passive viewing experiment (Wilke et al., 2009). The authors suggested that this might be related to the role of the pulvinar in attention, decision-making, or behavioral planning.

According to Tsuchiya et al. (2015), this finding illustrates that the report paradigm can lead to the misattribution of neural signals to conscious experience. However, this could be questioned. A notable asymmetry between both experiments is that in the report condition, stimuli were ambiguous, and in the no-report control condition they were not. An effect of ambiguity in the beta band of EEG was observed in yet another analysis of Nikolaev et al. (2010). The more ambiguous the stimulus, the more reduced the stimulus-evoked synchrony in the beta band (around 20 Hz). These evoked synchronies were observed over occipital and occipito-parietal regions of the cortex, above the extrastriate areas from which the pulvinar receives extensive input. Nikolaev et al. (2010) interpreted the evoked synchrony as facilitating the global broadcasting of visual information. The less ambiguous the stimulus the more stimulus information there is to be broadcasted, and so the longer the duration of the synchrony. In Nikolaev et al. (2010), the evoked synchrony arose earlier and lasted shorter than in Wilke et al. (2009), and the effect of ambiguity was the opposite. This might well be a result of the difference in stimuli or task. However, it will be clear that stimulus ambiguity can be a confounding factor in the comparison between report and no-report conditions.

#### No-Report Binocular Rivalry With Nystagmus

Studies of binocular rivalry using the report paradigm found activation in the prefrontal and parietal cortex, which led researchers to conclude that higher-order processes are essential for a conscious experience (e.g., Sterzer et al., 2009). Frässle et al. (2014) challenged this idea by proposing that the use of subjective reports was responsible for the prefrontal activation, not the conscious perceptual switches (Michel, 2017). To test this assumption, they used a no-report version of the binocular rivalry paradigm. Instead of reports, they employed a phenomenon called Optokinetic nystagmus (OKN). OKN occurs in eye movement relating to the pursuit of a moving stimulus. It consists of two alternating phases. In the slow phase, OKN follows the perceived direction of the movement. In the fast phase, the eye saccades back in the opposite direction to reset the eye position (Leopold et al., 1995).

When an observer is presented with stimuli moving in opposite directions in each eye, perceptual rivalry occurs. One of the rivalrous movement directions will become perceptually dominant while the other is suppressed. Which one is dominant can be inferred from the directions of the slow pursuit and fast reset of OKN (Frässle et al., 2014).

**Figure 2:**
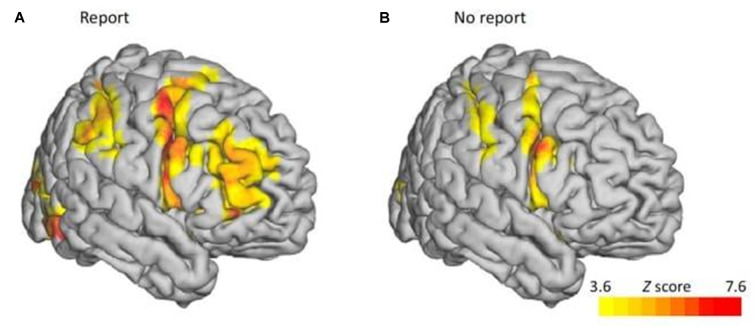
fMRI results of Report vs. No-Report Binocular Rivalry. fMRI blood oxygen level-dependent (BOLD) contrasts between perceptual switches and yoked stimulus during binocular rivalry revealed lower activity in the prefrontal cortex in the report condition **(A)** compared to the no-report condition**(B)**. Adapted with permission from the publisher from “No-Report Paradigms: Extracting the True Neural Correlates of Consciousness,” by Tsuchiya et al. (2015). Copyright 2015 by Cell Press.

After demonstrating that OKN correlates with self-reports of perceptual dominance, Frässle et al. (2014) compared a condition, in which the perceived direction was reported, with a no-report one, in which it was inferred from OKN. As shown in Figure 2, they found that activity related to switching was less intense overall in the no-report condition compared to the report condition. In particular, bilaterally the activation in the middle frontal gyrus completely disappeared in the no-report condition. This led the researchers to conclude that this activity was unlikely to be driving the switching behavior. Rather, it is associated with neural consequents such as monitoring and evaluation in preparation for the report.

The middle frontal gyrus is a region where dorsal and ventral attention networks converge and is believed to control the switch from the dorsal ongoing endogenous attentional processes to an exogenous stimulus (Japee et al., 2015). It belongs to a subsystem of the Default Mode Network (DMN). The DMN is related to a broad range (Kawagoe and Kase, 2021) of inward experiences (Spreng et al., 2009); in particular, the middle frontal gyrus subsystems have been preferentially related to thinking about the future self (Xu et al., 2016). This observation allows an alternative interpretation of the results of Frässle et al. (2014). It might be a mistake to think that by eliminating the report, the mechanisms of conscious experience keep doing exactly the same. Presumably, when the task changes to passive viewing, we become less involved in the stimulus; it becomes boring and monotonous, and the mind starts to wander (Smallwood and Schooler, 2006). Mind wandering and daydreaming are related states that can arise spontaneously and are characterized by a peculiar dynamic that allows thought to turn inward (Christoff et al., 2016). According to this dynamic, spontaneous mind wandering can alternate with periods of focused attention over the course of intervals of variable length (Nakatani et al., 2019, 2021). This may be the reason why contrasts are attenuated in the no-report paradigm (Barron et al., 2011). One’s future self is one of the subjects that feature prominently in mind wandering and daydreaming (Stawarczyk et al., 2013). In absence of report, the middle frontal gyrus may have changed its role from controlling switching to controlling the switching, or time-sharing, between attention to the task and the ongoing inner experience. This is why the contrast with replay in Frässle et al. (2014) disappears. In other words, the disappearance of the effect may not be a sign that the medial frontal gyrus is a mere neural consequent in conscious experience, but rather a sign that consciousness in the no-report condition finds an occupation elsewhere. In other words, it is important to realize what major reorientations take place in consciousness between report and no-report conditions, namely a disengagement from the task.

#### Inattentional Blindness

To avoid the problems involved in a direct comparison between report and no-report conditions, Pitts et al. (2014) adopted an inattentional blindness paradigm consisting of three phases. In Phase one, participants performed a distractor task while a critical but unexpected stimulus was also presented. Following this phase, the participants were questioned whether they had been aware of the critical stimulus (inattentional blindness was found in at least half the participants). In Phase two, due to the intervening questions asked, the participants were aware of the critical stimulus but still performed the distractor task. In the third phase, participants were asked to attend to the critical stimulus and perform a task associated with it.

Contrasting EEG recordings from the first and the second phase (awareness vs. unawareness) should reveal NCC. On the other hand, contrasting the neural activity of the second and the third phase should reveal activity related to reporting. Previous EEG research using report-based paradigms found increased gamma-band activity as well as the P3 component (a late component of ERP observed 300 ms after an event) associated with consciousness (e.g., Batterink et al., 2012). These findings turned out robust only for the second comparison and not the first. This led the researchers to conclude that such neural activity is related to reporting and does not identify an NCC (Pitts et al., 2012, 2014).

These findings, however, should not surprise anyone, given the extraordinary task-specificity of EEG. The same task was used in Phases 1 and 2; a different one in Phase 3. Now the intended contrast between Phases 2 and 3 is obviously report/no-report. But there are confounding differences between these phases, such as: target identity, target history (e.g., the fact that the target of Phase 3 may have lost the salience it had in Phase 2), and time on task; comparison of the first two phases may suggest that the effects in ERP and gamma are not selective to consciousness; any comparison with Phase 3, however, is inconclusive because of confounding.

#### No-Report Binocular Rivalry With a Fixation Task

A close-up view of the studies highlighted in Tsuchiya et al. (2015) as exemplars of the no-report paradigm reveals that none of these studies unambiguously demonstrates that frontal activation in conscious experience is a confound of reporting the percept. In the years since these seminal studies were published, the no-report paradigm has evolved further, which has alleviated some of the concerns. As a recent example, consider Hesse and Tsao’s (2020). In a binocular rivalry, macaques were continuously presented with a taco image in one eye and a face image in the other. To eliminate the report, a fixation task was used. A fixation cross was presented at one of the four corners of each image. The fixation cross jumped between the corners, differently for the face image and the taco image. Hence, the dominant percept could be identified, based on which fixation cross was tracked. In the physical condition, the monkeys were monocularly presented with alternations of the face and the taco image. The researchers collected recordings from a novel 128-electrode site probe in the macaques’ inferior temporal cortex. Mainly they focused on the middle lateral (ML) and the anterior middle (AM) regions, also known as face patches. Their observations show that neuronal activity is modulated by the percept. With this, they arguably show a correlate of consciousness as envisaged by Koch (2004). The result is not affected by previous issues relating to disengagement from the task, as tracking the fixation cross is engaging in its own right. However, their results also show that neuronal activity is modulated by both the percept and the physical stimulus. In fact, the latter modulation is stronger than the former. This suggests, consistently with Wilke et al. (2009), that binocular rivalry is not completely resolved at the level of the inferotemporal cortex (IT; cf. Zou et al., 2016). Authors write: “It remains an open question where and how the conscious percept is ultimately isolated from the suppressed stimulus to produce conscious awareness of the former and not the latter.” We may comment: the current no-report paradigm does not exclude a role for the frontal system in this process.

## Contribution of The No-Report Paradigm

We have seen researchers use inventive designs to overcome the problem of how to identify the percept without report. The no-report approach envisaged demonstrating that certain processes that are correlated with consciousness are, in fact, not NCC but preconditions or consequences. They claimed that these are confounded in NCC because they are elicited by the need to report.

To show this it is necessary to compare report and no-report conditions. However, this turns out more difficult than expected. We discussed several examples brought forward by Tsuchiya et al. (2015) to show contrasts between both paradigms; in particular regarding the involvement of the prefrontal cortex (Block, 2019). We found alternative explanations and/or serious methodological flaws in all these studies. The case, in our opinion, is far from closed.

### Limitations of the No-Report Paradigm

If no gain can be won from a direct comparison between report and no-report conditions, let us consider the merits of the no-report paradigm in its own right. First of all, how well might it be expected to do on its own terms, in highlighting NCC at the expense of their neural prerequisites and consequences?

#### No-Report Paradigms May Still Be Over-Inclusive

No-report may not be free from post-NCC neural activities as consciousness. Even if a participant is not asked to report what they see, they might not refrain from giving attention to the stimulus, introspecting on their experience, or developing a meta-cognitive awareness. Block (2020) called this the “bored monkey” problem, suggesting that this is because participants have too little to do.

To overcome this problem, Block (2019) suggested a *no-cognition* paradigm. As a variant of the no-report paradigm, this paradigm involves presenting stimuli that are perceived but do not draw attention, and thus do not give rise to post-perceptual processes. As an example, he cites Brascamp et al. (2015), a study in binocular rivalry presenting moving dots to both eyes, part of which move coherently in orthogonal directions between both eyes. Frequent switches in the actual physical movement direction of the dots reduce the saliency of switches in eye dominance. This reduced the effect of switches in dominance in frontal areas compared to the condition where the switch was conspicuous. Whereas this may show that the switches are not initiated in the frontal areas, it does not speak against their involvement in their conscious awareness (Philips and Morales, 2020). As we have seen with switches, they can be caused by sensory or oculomotor events. All it shows is that their conscious perception does not differ from similarly inconspicuous changes that are sensory in origin, as they occur in the stimulus.

While it could be argued that Brascamp et al.’s (2015) study evaded the “bored monkey” problem, there is another problem that remains unaddressed. We encountered this problem in our detailed account of the No-Report- Binocular Rivalry with Nystagmus. Namely, without a task, the participant is likely to disengage from the stimulus and engage in mind wandering or daydreaming Mind-wandering and daydreaming are states that arise spontaneously (Christoff et al., 2016) in a dynamic that alternates with focused attention to the stimulus. Thus, the effects observed in the no-report paradigms maybe something else than NCC.

Most importantly, it is not sufficient to compare report and no-report conditions by just eliminating the report, keeping everything else constant. Manipulating one factor at a time is a good practice of experimental design, but here, this just is going through the motions. In fact, eliminating report from the task dramatically changes the cognitive load, and makes the task less engaging. Consciousness is fickle; in case a task is less engaging the mind will find other things to do. Mind-wandering and daydreaming will inevitably creep in. As a result, the contrasts between report and no-report, rather than revealing NCC, may reveal the neural correlates of mental disengagement and a mind turned inward.

In addition, the no-report paradigm may still be overinclusive regarding unconscious neural processing (Tsuchiya et al., 2015). The incorporation of unconscious neural processing in NCC is particularly relevant in binocular rivalry studies, a prime example of the no-report paradigm. However, the switches that are measured and aimed to reflect conscious perception changes can actually be found in several different stages of information processing (Frässle et al., 2014). Studies show that pupil size and the associated eye movement can also be seen in pre-conscious events (Vandenbroucke et al., 2014; Firestone and Scholl, 2016). Moreover, the results obtained through binocular rivalry experiments using no-report paradigms do not converge. Frässle et al. (2014), which we discussed, found that reports matched the objective eye movement measures while the differential neural activity of frontal areas was observed. On the other hand, Kapoor et al. (2018) and Kapoor et al. (2019) investigating electrophysiological signals in prefrontal areas of monkeys, found that prefrontal cortex activation, namely neuronal discharges, were “robustly modulated in accordance with the animal’s conscious perception”. Further, they found that the spiking activity elicited was similar to this perceptual modulation’s strength. They concluded that the prefrontal activity during a no-report binocular rivalry paradigm was related to consciousness in visual perception.

Finally, binocular rivalry not only occurs in visible stimuli but also has been shown in invisible stimuli in humans (Zou et al., 2016). Many animals including species that are not closely related to humans such as fruit flies (Miller et al., 2012) have been shown to act in resemblance to rivalry-like state changes in humans. Consequently, the binocular rivalry is not purely a conscious process (Aru et al., 2012), and is not a dependable measure of consciousness when used single-handedly.

#### No-Report May Be Underinclusive

Besides the issue of overinclusion, no-report paradigms may also be underinclusive. That is, some concepts, particularly abstract ones that lack a perceivable referent, may not become conscious before an attempt is made to express them (Kiefer and Pulvermüller, 2012; Dove, 2016). Are we really aware of something abstract, like love or modesty, without verbalizing (Paivio, 1990) or imaging it? Even if a stimulus is sensed, sometimes it may only be possible to fully perceive consciously what we have registered through post-perceptual processes (Kemmerer, 2015). If the participant is shown some unknown vehicle and then is asked: “Did you see the Terrafugia?”, this will induce a post-perceptual interpretation process that will disambiguate and retrospectively enrich the content of the experience. Are we justified in excluding the retrospective element from NCC, or is the experience incomplete without it? In this respect, it is relevant that, for instance, Hesse and Tsao’s (2020) no-report paradigm confirms that the selection of the percept is incomplete at an earlier visual level. Therefore, it is possible for no-report paradigms to include NCC as well. In sum, no-report paradigms were conceived as an attempt to deal with the problems of overinclusiveness of the report paradigms. Despite their promise, they face their own unique challenges.

## Conclusion

Opinions differ as to which neural mechanisms and processes are essential for consciousness. Researchers promoting a restricted view have pushed for no-report paradigms to clear their results of, what they believe, are confounds. We have taken a closer look at the results of no-report paradigms brought forward to show that certain processes, especially in the prefrontal cortex, are non-essential. We found these results unconvincing, beset with alternative explanations and methodological flaws. No-report paradigms tend to be less engaging than report paradigms, and therefore attenuate the signal. The disengagement of pre-frontal cortex could be understood as a qualitative change in consciousness, in which it turns inward and engages in daydreaming and mind-wandering. Considered on its own terms, no-report paradigms could still fail to eliminate spurious correlates, either because these mechanisms are actually necessary or whether they are deployed habitually. People (and other animals) may pay attention to the stimulus and reflectively observe their experience, even when they’re instructed not to do so, as an absence of a report does not automatically make participants passive subjects (Block, 2019).

On the other hand, the use of no-report paradigms to investigate consciousness is still fairly new and it remains to be seen if further developments can identify a limited set of NCC. The challenge posed by the restrictive view on NCC is still open. All neuronal research is based on certain preconceptions about the role of processes like attention and self-reflection in conscious experience and access to content (Schlicht, 2018). As McGinn (1989) put it, “we know that brains are the *de facto* causal basis of consciousness, but we have, it seems, no understanding whatever of how this can be so” (p. 349). This may still be as true to date.

## Data Availability Statement

The original contributions presented in the study are included in the article, further inquiries can be directed to the corresponding author.

## Author Contributions

ID, IE, AG, ZG, and JB completed a version of this text as a writing assignment in the framework of the advanced methods course within the Department of Psychology and Educational Sciences of the KU Leuven. They all contributed equally to the article under the supervision of CL, who revised the article and prepared the submission. All authors contributed to the article and approved the submitted version.

## Conflict of Interest

The authors declare that the research was conducted in the absence of any commercial or financial relationships that could be construed as a potential conflict of interest.

## Publisher’s Note

All claims expressed in this article are solely those of the authors and do not necessarily represent those of their affiliated organizations, or those of the publisher, the editors and the reviewers. Any product that may be evaluated in this article, or claim that may be made by its manufacturer, is not guaranteed or endorsed by the publisher.
